# Navigating work after prostate cancer treatment: a systematic review of qualitative research

**DOI:** 10.1007/s11764-026-02062-w

**Published:** 2026-06-16

**Authors:** Dunya Tomic, Tessa Keegel, Sophie K. Schellack, Lisa Abdel-Malek, Caron Jander, Supun T. Hettige, Ewan MacFarlane, Jeremy Millar, Karen Walker-Bone

**Affiliations:** 1 Monash Centre for Occupational and Environmental Health, School of Public Health and Preventive Medicine, Monash University, 553 St Kilda Road, Melbourne, VIC 3004 Australia; 2 German Cancer Society, Kuno-Fischer-Straße 8, 14057 Berlin, Germany; 3 Monash University, Wellington Road, Clayton, VIC 3800 Australia; 4 Alfred Health, 55 Commercial Road, Melbourne, VIC 3004 Australia; 5 School of Translational Medicine, Monash University, 99 Commercial Road, Melbourne, VIC 3004 Australia

**Keywords:** Prostate cancer, Cancer survivorship, Work participation, Return to work, Qualitative synthesis, Systematic review

## Abstract

**Purpose:**

Prostate cancer treatment can disrupt working life, yet there is limited understanding of how men experience work during and after treatment. This review aimed to synthesise qualitative evidence on prostate cancer survivors’ work-related experiences.

**Methods:**

We searched Ovid MEDLINE, Embase, PsycINFO, CINAHL, and Google Scholar for qualitative research reporting men’s work experiences during or after prostate cancer treatment. Screening, quality appraisal, and thematic synthesis followed PRISMA guidelines and the Joanna Briggs Institute Critical Appraisal Tool. The review was registered on PROSPERO (CRD420251083779).

**Results:**

Eleven studies involving 302 men were included. Six themes emerged: return-to-work timing and trajectories; financial pressures; support systems; treatment side effects and stigma; adjustments and adaptations; and work identity, values, and priorities. Men described variable return-to-work pathways, influenced by treatment effects such as incontinence. Workplace and family support helped facilitate work participation, while men adapted responsibilities to accommodate health needs. Work held multifaceted meaning, serving as a source of identity and coping, although some men reprioritised their identities toward health and family goals.

**Conclusions:**

Men’s return to work after prostate cancer is shaped by treatment effects, workplace flexibility, and social support, within the workplace and from family and community support services. Evidence on interventions to support reintegration is limited, highlighting the need for tailored occupational and survivorship strategies.

**Implications for cancer survivors:**

Returning to work involves balancing personal health, workplace demands, and social responsibilities. Survivors can benefit from proactively communicating needs to employers, seeking tailored workplace adjustments, and leveraging support networks to sustain meaningful work participation.

**Supplementary Information:**

The online version contains supplementary material available at https://doi.org/10.1007/s11764-026-02062-w.

## Introduction

Prostate cancer is often viewed as a manageable disease, due to its high survival rates and relatively slow progression in many cases [1]. Yet this framing can obscure the often-disruptive consequences of treatment, especially for those diagnosed during their working lives [2]. While the majority of prostate cancer diagnoses occur in older men, estimates suggest around 10% of new diagnoses occur before the age of 55 [3]. Even among older men, it is increasingly common for many to remain in the workforce beyond traditional retirement age, amid shifting economic conditions and evolving expectations around ageing, productivity, and work [4]. In this context, navigating employment following prostate cancer treatment is not just a matter of physical recovery or financial need but often involves renegotiating identity, role expectations, and social relationships. Treatments such as surgery, radiotherapy, and hormone therapy can affect energy levels, continence, and cognitive focus [5], which may influence men’s capacity to perform work and engage confidently with colleagues, clients, and employers. These impacts are not easily captured through biomedical endpoints or standard measures of workforce participation.

Qualitative research offers lived-experience insights about how men experience the intersection of cancer, treatment, and working life—not simply in terms of whether they return to work, but how they make sense of these experiences, what they value, and how their choices are shaped by workplace culture, gender norms, healthcare interactions, and personal networks [6]. Work may represent routine, purpose, and a return to normalcy—or conversely, a source of strain or a threat to wellbeing [7]. These meanings can shift over time and may differ by employment type or cultural contexts [8]. While qualitative studies have begun to explore these issues in relation to prostate cancer treatment and work [9–11], to date, there has been no systematic synthesis of the qualitative literature. This review therefore aims to synthesise qualitative research about how men experience work in the context of prostate cancer treatment and recovery, to inform person-centred approaches to survivorship support across clinical and occupational settings.

## Methods

### Search strategy

A comprehensive search strategy was developed in collaboration with an Information Scientist. On May 27, 2025, searches were performed across five databases: Ovid MEDLINE, Embase, PsycINFO, CINAHL, and Google Scholar (first 50 references). Google Scholar was searched to identify additional peer-reviewed studies not indexed in traditional databases, using a pragmatic limit of the first 50 results sorted by relevance, consistent with approaches used in prior systematic reviews [12]. The search combined terms related to prostate cancer and work participation using both keywords and database-specific subject headings (MeSH, EMTREE) (Supplementary Table 1). Retrieved records were imported into EndNote for duplicate removal and then uploaded into Covidence for screening and management. Searches were conducted without language restrictions; however, only English-language studies were included at the screening stage due to the need for consistent interpretation during independent dual screening of qualitative studies and because all reviewers were fluent in English.

### Inclusion and exclusion criteria

The inclusion criteria were guided by the PICo framework [13] (population = men offered treatment for prostate cancer; phenomenon of interest = experiences of work during or after treatment; context = work participation, including employment, return to work, work adjustments, or challenges). Eligible studies were those reporting original qualitative data or mixed-methods research with extractable qualitative findings. Only studies published in English that reported prostate cancer treatment types received by the participants were included. Studies were excluded if they did not specify treatment type, were quantitative-only studies, or lacked qualitative data on work experiences. Other exclusions included reviews, meta-analyses, editorials, commentaries, conference abstracts, and dissertations. Reference lists of relevant reviews were manually searched for additional eligible studies.

### Review protocol and data extraction

This review followed the PRISMA guidelines [14] (Supplementary Table 2) and the ENTREQ checklist [15] (Supplementary Table 3) and was registered prospectively with PROSPERO (registration number CRD420251083779). Title/abstract and full-text screening were conducted independently by two reviewers. Inter-rater agreement was high, with proportionate agreement of 91–92% at the title/abstract stage (Cohen’s *κ* = 0.41–0.52) and 98–100% at the full-text stage (Cohen’s *κ* = 0.96–1.00). Discrepancies were resolved through discussion, with involvement of a third reviewer where required.

A standardised data extraction form was developed and piloted on two included studies before being applied to the full set (Supplementary Table 4). Data were extracted into a structured Microsoft Excel spreadsheet by one reviewer (DT, TK, or SKS) and independently checked by a second reviewer, with discrepancies resolved through discussion. Extracted information included author, year, source, country, sample size, participant characteristics, treatment details, data collection methods, data collection timing, qualitative analytic approach, and work-related findings. For mixed-methods studies, only the qualitative components (e.g. participant quotes and authors’ thematic interpretations) were extracted and analysed. The results and discussion sections of each included study were reviewed line by line, and all qualitative data relating to participants’ work experiences during and after prostate cancer treatment were extracted, including verbatim participant quotes and author-reported themes.

Microsoft Excel was used for data extraction, coding, and thematic synthesis. Excel enabled structured tabulation of extracted data, codes, and corresponding quotes within a single transparent workspace, supporting independent checking and collaborative codebook development. Given the volume of qualitative data and the team’s familiarity with the software, Excel provided a practical and reproducible workspace for the synthesis.

### Risk of bias assessment

Risk of bias was assessed using the Joanna Briggs Institute critical appraisal checklist for qualitative research [16]. An overall rating of low, moderate, or high risk of bias was assigned based on the number and nature of domains rated “No” or “Unclear”. Studies were rated low risk when all core methodological domains (Q1–Q5, Q8, Q10) and ethical conduct (Q9) were rated “Yes”, with concerns limited to researcher reflexivity and cultural/theoretical positioning (Q6, Q7). Studies were rated moderate risk when they had additional “Unclear” or “No” ratings beyond Q6 and Q7, for example, relating to philosophical perspective (Q1) or ethical reporting (Q9), but where these did not compromise the overall methodological validity or interpretability of the findings. Studies were rated high risk if they exhibited multiple “No” ratings across core methodological domains (Q2–Q5) or if critical concerns about ethical conduct (Q9) or representation of participants’ voices (Q8) were identified. Risk of bias ratings were used to guide the interpretation of findings but did not determine study inclusion. Formal sensitivity analyses re-synthesising the data with higher-risk studies excluded were not conducted, as this approach is generally less informative for qualitative synthesis than for quantitative meta-analysis. Instead, the influence of study quality on confidence in each analytical theme was considered during interpretation.

### Data synthesis

Thematic synthesis, as outlined by Thomas and Harden [17], was used to synthesise qualitative findings. One reviewer (DT) conducted line-by-line coding of relevant text from the included studies in Excel, generating descriptive codes inductively from participants’ accounts and authors’ interpretations. Codes were independently checked by two further reviewers (TK and SKS), and all three reviewers met to discuss and finalise the codebook through iterative discussion. Codes were grouped into descriptive themes and subsequently developed into analytical themes to provide deeper interpretive insights into men’s experiences navigating work during and after prostate cancer treatment. Coding continued until no new codes or thematic dimensions emerged from additional studies, indicating that the synthesis adequately captured the range of experiences described in the included literature. A worked example illustrating the progression from quote to code to descriptive theme to analytical theme is provided in Supplementary Table 5.

### Survivor involvement

Two prostate cancer survivors were invited to review the synthesised findings to ensure clarity and resonance with lived experience. They were identified through the professional and personal networks of the authors and were approached by email with a copy of the manuscript. Feedback was provided via email and through informal one-to-one discussions with two members of the research team (KWB and DT). Both survivors had received treatment for prostate cancer, and one was undergoing active treatment at the time of feedback. Their input prompted minor revisions to wording and informed the framing of the discussion.

## Results

**Fig. 1:**
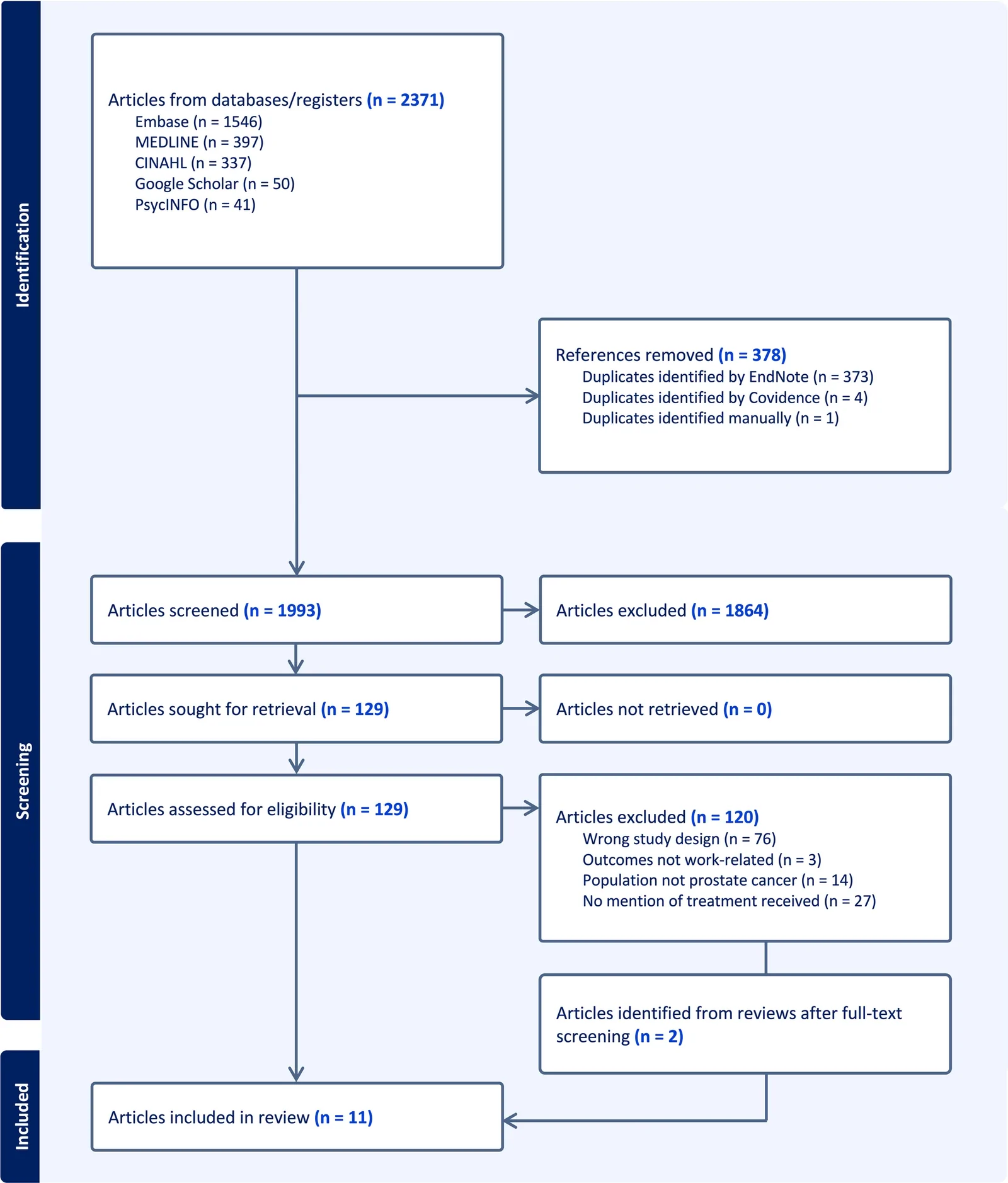
Preferred reporting items for systematic reviews and meta-analyses (PRISMA) flow diagram

After removal of duplicates, 1993 records were screened at the title and abstract level. A total of 129 full-text articles were assessed for eligibility, of which 120 were excluded (Fig. 1), most commonly due to incorrect study design, lack of relevant work-related outcomes, or population not restricted to prostate cancer (e.g. studies including mixed cancer populations without extractable prostate cancer-specific data). An additional two articles were identified through reference list screening, resulting in 11 studies included in the review [9–11, 18–25]. These studies reported qualitative data on the work-related experiences of 302 men who had undergone treatment for prostate cancer (Table 1). Most were conducted in the UK (*n* = 4), the USA (*n* = 3), or Canada (*n* = 2), with one study each from Australia and Sweden. Work-related experiences were the focus of 5/11 studies, while the remaining studies examined topics such as everyday life following treatment, adjustment strategies, and financial difficulties. Two studies only included men treated with radical prostatectomy (RP), one only those who received external beam radiation therapy (EBRT), while most samples included men who received various treatments including RP, radiotherapy, hormone therapy, chemotherapy, and/or active surveillance (AS)/watchful waiting (WW). Occupation was reported in 5/11 studies, generally in broad terms such as white-collar vs. blue-collar or professional vs. manager.

**Table 1:** Characteristics of the 11 included studies exploring work-related experiences among men treated for prostate cancer

Author, year	Country	Study focus	*N* (prostate cancer)	Treatment type(s)	Participant characteristics	Data collection method	Data collection time	Analysis method
Al Hussein Al Awamlh 2024 [10]	USA	Early and long-term lived experiences after prostate cancer treatment	66	AS (33%), radiotherapy (30%), surgery (36%)	Age: all < 80 years (18% < 55, 82% 55–79) Ethnicity: White 76%, Black 14%, Hispanic 2%, Asian 2%, Native American 2%, other 3%, NS 3%) Employment: many retired or near retirement at diagnosis (proportion NS) Occupation: NS	Semi-structured interviews	10 years post-diagnosis	Constant comparative analysis (grounded theory)
Appleton 2015 [18]	UK	The impact of prostate cancer on men’s everyday life	27	EBRT combined with surgery and/or hormone therapy	Age: 57–76 years Ethnicity: NS Employment: 22/27 retired at interview Occupation: NS	Semi-structured interviews	Before radiotherapy, 6–8 months after and 12–18 months after	Constant comparative analysis (grounded theory)
Gray 2005 [19]	Canada	Life experiences of Black men with prostate cancer	2	Radiotherapy (50%), surgery (50%)	Age: 62 and mid-70s Ethnicity: both Black Employment: one employed, one retired Occupation: factory work	Unstructured interviews	Minimum 1-year post-diagnosis	Narrative analysis
Grunfeld 2013 [9]	UK	Work after treatment for prostate cancer	50	Chemotherapy, combined with surgery (66%), radiotherapy (6%), or brachytherapy (28%)	Age: 18–64 years, mean 59 Ethnicity: White 76%, Black 18%, Asian 3% Employment status: all in paid employment at diagnosis; full-time 80%, self-employed 22% Occupation: White-collar 56%, blue-collar 30%, pink-collar* 14%	Semi-structured interviews	Within 1 month of treatment and again 12 months post-treatment	Framework analysis
Joyce 2024 [20]	USA	Financial toxicity among men diagnosed with prostate cancer	35	Chemotherapy, radiotherapy, surgery	Age: median 69 years Ethnicity: White 94%, Black 3%, other 3% Employment: retired 71%, full-time 14%, part-time 9%, self-employed 3%, not working outside the home 3% Occupation: NS	Semi-structured focus groups	NS	Thematic analysis (informed by financial capacity models)
Liu 2023 [21]	Australia	Work-related experiences of prostate cancer survivors	16	Combinations including surgery (63%), hormone therapy (38%), chemotherapy (25%), radiotherapy (50%), AS (6%)	Age: 55–81 years, median 68 Ethnicity: NS Employment: at diagnosis salaried 38%, self-employed 62%; at interview retired 63%, working 25%, unemployed 13% Occupation: professional 63%, manager 19%, trade worker 13%, technician 6%	Semi-structured interviews	Range from < 1 year to 19 years post-diagnosis	Inductive thematic analysis
Owens 2024 [11]	USA	Experiences of work among prostate cancer survivors	45	RP (53%), radiotherapy (38%), both (9%)	Age: 46–78 years, median 68 Ethnicity: White 56%, Black 44% Employment: all employed at treatment (inclusion criterion) Occupation: manager or professional 60%, agriculture, construction, production, transportation 22%, sales and office 11%, service 7%	Semi-structured interviews	6–36 months post-treatment	Grounded theory-informed content analysis
Wagland 2020 [22]	UK	Adjustment strategies after prostate cancer treatment	14	RP (43%), radiotherapy/ADT (29%), ADT only (7%), AS (7%), WW (7%), chemotherapy (7%)	Age: 55–85 years, median 66 Ethnicity: all White Employment: 10/14 employed, 4/14 retired Occupation: NS	Semi-structured interviews	18–42 months post-diagnosis	Inductive framework analysis
Wennick 2017 [23]	Sweden	Experiences of everyday life after RP	19	RP	Age: 49–65 years, median 62 Ethnicity: NS Employment: NS Occupation: NS	Semi-structured interviews	12–18 months post-RP	Inductive thematic content analysis
Yarker 2010 [24]	UK	Role of communication and support in RTW after cancer	4	Surgery (50%), radiotherapy/hormone therapy (25%), other (25%)	Age: 61–63 years Ethnicity: NS Employment: 3/4 employed, 1/4 unemployed Occupation: security officer, postman, administrative technician	Semi-structured interviews	Up to 3 years post-diagnosis	Inductive thematic analysis
Yu Ko 2020 [25]	Canada	RTW after RP and connection to masculinity	24	RP	Age: all ages (13% < 55 years, 17% 55–60, 29% 61–64, 42% ≥ 65) Ethnicity: NS Employment: all employed at diagnosis; 12/24 self-employed, 12/24 employees Occupation: NS	Semi-structured interviews	Up to 3 years post-RP	Constructivist grounded theory with masculinity theory

*ADT* androgen deprivation therapy, *AS* active surveillance, *EBRT* external beam radiation therapy, *NS* not stated, *RP* radical prostatectomy, *RTW* return to work, *WW* watchful waiting

*In Grunfeld 2013, participants working in clerical, sales, and service occupations were classified as “pink-collar”

### Risk of bias assessment

Of the 11 included studies, nine were rated at moderate risk of bias, and two at low risk (Supplementary Table 6). No studies were assessed at high risk of bias. There was universally low risk of bias across most categories (Supplementary Fig. 1). However, only two studies clearly articulated a philosophical perspective, only one included a cultural or theoretical location statement, and none addressed the influence of the researchers on the research or vice-versa. Additionally, several studies did not clearly report ethics approval.

### Thematic synthesis results

Ten descriptive themes were developed inductively and integrated into six analytical themes (Table 2): (1) return-to-work timing and trajectories; (2) financial pressures and economic trade-offs; (3) support systems; (4) treatment side effects and stigma; (5) adjustments and adaptations; and (6) work identity, values, and priorities. Direct quotes from study participants are indicated in italics using quotation marks; quotes from study authors are presented in italics without quotation marks.

**Table 2:** Analytic and descriptive themes on work and prostate cancer survivorship

Analytic themes (studies included in themes as citations)	Descriptive themes	Quote (examples)
Support systems [11, 18–21, 23–25]	Employer and organisational support; communication, disclosure, and stigma; support systems and relationships	*“I’m on shifts but I arranged with work so I could go in and stay on the morning shifts so I would go in to work for 6 o’clock, finish at 10 o’clock, come home get changed go to.... and have the treatment”*. [18]
Work identity, values, and priorities [9, 11, 21–23, 25]	Work as identity, coping, and motivation; values, priorities, and retirement decisions	As one man explained, *“(My wife and I) live comfortably and don’t have any major expenses. (That’s why) I thought about this for a long time and felt that I should retire if the conditions (at work) were right. Instead, I could spend more time with my family or be doing things I enjoy”*. [25]
Treatment side effects and stigma [9–11, 20, 21, 25]	Treatment side effects and their impact on work; communication, disclosure, and stigma	*“I’m going to be constantly backward and forward to the toilet checking that everything’s OK and they’re [employer] going to do their nut about it. So, I’d rather not go back in that aspect until I know I’m going to be fairly reasonable when I get back to work”*. [9]
Adjustments and adaptations [11, 21, 24, 25]	Employer and organisational support; work adjustments and role flexibility	*“I was on a van route, but it was a long day, and I asked to be put on a shorter route”*. [24]
Return-to-work timing and trajectories [9, 10, 20, 21, 25]	Return-to-work timing and trajectories; self-employment: autonomy and risk	*“If I stop work that means marketing isn’t done which means work doesn’t come in. It’s not as if I had a choice of diving into work at the beginning (after the surgery), the choice wasn’t there. It was me doing more marketing type stuff, which is still work ….”* [21]
Financial pressures and economic trade-offs [9, 10, 20, 21, 25]	Financial pressures and economic trade-offs; self-employment: autonomy and risk	*“I’ve been with [company] for 35 years, but I was in a status where I didn’t have benefits. [The administrator] said, ‘So, what we’re going to do is we’re going to pay you for all your time off anyway. We just feel that’s the right thing to do’. And took it out of the [company’s] budget. So, I got great support from everybody”*. [10]

### Return-to-work timing and trajectories

Men described highly variable pathways back to work following prostate cancer treatment. For some, the impact was minimal: *“I missed a few days initially when I had the treatment, but other than that it doesn’t affect my ability to do my job”* [10]. Others, meanwhile, experienced longer absences than expected: *“For the second surgery, the recovery was much more difficult than they led me to believe. They were saying I can go back to work in like a week or two, that [ended up] being more like six weeks”* [10]. For some, continuing to work was not a matter of choice but of circumstance, particularly among those who were self-employed: *“If you were to ask me if I were employed by someone would I be back at work. I guess if I wasn’t self-employed, I wouldn’t be at work. But I am self-employed, and I always have been, so when I get a cold or sore throat my first thought isn’t ‘I’ll stay in bed today’. I’ve got to go to work”* [9]. Others highlighted the necessity of keeping business activity alive even during recovery: *“If I stop work that means marketing isn’t done which means work doesn’t come in. It’s not as if I had a choice of diving into work”* [21].

Some men described intermittent or staggered participation in work, often linked to changing demands of treatment: *“I did the [first] work for about eighteen months. I took a break for about six months and then I got the opportunity to [do the second work] … I’d had a break from the hormone therapy at the time I started [the second work], so I was sort of feeling a lot better… When I finished the project and I’d had to go back on the medication that I felt like I really sort of hit the wall and went into a bit of a slump”* [21]. Others reported a gradual expansion of workload over time, such as one who noted that initially he *“could only work for a few hours a day”*, but later, when surgical wounds healed, *“I started working eight or more hours a day”* [25]. These narratives also emphasised the ongoing process of evaluating one’s own capacity: *“I was feeling stronger by the fifth or sixth week (after returning to work) and I thought I could start working on the larger projects. But I didn’t tell my clients and decided to wait until I was totally certain that I could handle the work”* [25].

Work, however, was rarely discussed in pre-treatment consultations, leaving some men unprepared for the trajectory ahead: *“No [discussion about work], not really, it was only advised to take it a bit easier with the lifting until things had healed properly”* [21]. Others expressed frustration at the lack of information about treatment side effects and their implications for work: *“I don’t feel like I was adequately informed around the impacts of treatment, particularly the hormone therapy … It affects your cognitive function so I felt like I couldn’t [work]”* [21].

### Financial pressures and economic trade-offs

The financial burden of prostate cancer treatment was shaped by employment type, access to benefits, and household responsibilities. While some men were able to manage costs through paid leave or employee assistance programmes, others faced significant economic strain. Those with adequate leave described relief: *“I was very fortunate that I had plenty of leave time”* [10], and employer-provided health insurance could protect against major financial hardship: *“I’ve maintained a job that provides me health insurance, which is the reason I could get the care that I did. Having health insurance, I have not had to suffer from this financially, hardly at all”* [10].

Men in commission-based or self-employed roles experienced more immediate financial consequences: *“I have the type of job where I get paid a base salary and commission, so obviously during the period of time that I wasn’t working, I wasn’t selling anything, so the commission side of my income went down”* [10]. Self-employed men often faced direct income loss: *“No work no pay… there’s not really any sick leave or annual leave, it comes out of my pocket either way”* [21], and some had to close businesses due to sudden health deterioration, with ripple effects for their households: *“So I sold my business, which is helpful having that money in the bank, but basically that was before retirement… So, my wife ended up working for longer than she really would’ve had to”* [20]. Financial pressures also influenced the urgency to return to work, sometimes overriding readiness or recovery considerations: *“I have a family, a mortgage and people to pay. I just had to come back [to work]”* [25].

### Support systems

Support systems emerged as central to men’s recovery and return to work. Employers were often described as going beyond formal entitlements to demonstrate loyalty and care, particularly for longstanding staff: *“I’ve been with [company] for 35 years, but I was in a status where I didn’t have benefits. [The administrator] said, ‘So, what we’re going to do is we’re going to pay you for all your time off anyway. We just feel that’s the right thing to do’. And took it out of the [company’s] budget. So, I got great support from everybody”* [10]. Others reported colleagues stepping in when health needs interfered with work: *“We were able to work around the times when I had my immune system down, I was able to get support from other people within the group”* [21]. Supportive reintegration into the workplace was also highlighted as meaningful: *“When I walked in, of course everyone was delighted to see me, I was welcomed back, and I was only there for two hours or so, and they joked, and things slowly built up”* [24]. Access to occupational health staff provided another avenue of reassurance: *“The occupational health nurse is a lovely person altogether. She told me that if I ever needed anyone to talk to, I was to go down to her”* [24]. For many, disclosure of their diagnosis, though initially daunting, became the gateway to greater understanding and support: *“Once you let people know, you get a lot more support”* [21].

Families played an equally pivotal role, providing emotional encouragement, assuming household responsibilities, and adjusting routines to protect men’s recovery. Several described their partners’ patience and determination: *“My wife from the first day, for the first second was, ‘we’re going to beat this’. She had a very strong attitude about it. She’s been very, very patient with me”* [20]. Community cancer services were also identified as *“a big help”* [21] in navigating financial supports, though some men felt that in local cancer support groups, *work-related issues or information were rarely discussed* [21]. Additionally, some reported frustration with disability job providers, describing *“extremely … no support from them”* in accessing meaningful assistance [21].

### Treatment side effects and stigma

Treatment side effects, particularly incontinence, shaped men’s experiences of returning to work and often carried a stigma that was more disruptive than the cancer itself. Coping strategies were sometimes invisible to colleagues, but privately burdensome: *“I had a catheter for six months, so that affected me at the time. I was able to work. I was working. My work schedule was as regular as ever. I just had a bag on my ankle, which nobody knew about”* [10]. For others, the need to manage pads or bags in social contexts highlighted a sense of social difference compared to colleagues: *“If you can imagine two or three blokes in a meeting, you take your comfort break, everyone trots off for a pee. They’re all going to these appalling looking urinals that blokes use. I’ve got to trot into a cubicle and change a pad. It’s kind of socially abnormal”* [9].

Other physical effects of treatment also interfered with both cognitive and manual work. Fatigue was frequently described as impairing decision-making capacity: *“The fatigue [is] like that, it’s not just physical, because it affects your mental processes as well. After a while you find you can’t think clearly, so you’re not in the right space for making proper management decisions”* [21]. Surgical wounds and dizziness also restricted tasks requiring strength or balance: *“I was concerned with lifting heavy objects because of the (surgical) wound. So, I couldn’t do that until it healed completely. But I also felt dizzy at times. That ruled out any work that required (climbing) the ladder or reaching high-up unless there was someone to help me”* [25].

Stigma extended beyond bodily symptoms to concerns about social perception and employability: *“You are thrown back into it and you wonder how you will be received, whether people will say ‘Here he comes, he’s been loafing off for three months’”* [9]. Others experienced overt reactions that made them feel different or stigmatised: *“When you get around the individuals, their mouth drops … their eyes get big, and they look at you. I’ll say, ‘Yeah, okay, I’ve got cancer. And I’ve dealt with that issue’. Let’s get over the… shock, [I’m] not damaged goods. I often felt like that when individuals would talk to me and look at me sometimes”* [11].

### Adjustments and adaptations

Men described a range of adjustments to accommodate treatment-related effects while maintaining work participation. Some arranged shifts around treatment schedules: *“I’m on shifts but I arranged with work so I could go in and stay on the morning shifts so I would go in to work for 6 o’clock, finish at 10 o’clock, come home get changed go to … and have the treatment”* [18]. Keeping employers informed allowed work to be scheduled around medical needs: *“I kept them up to date on the whole process … so it was all discussed with my boss, and they scheduled things around me”* [21]. For self-employed men, control over hours was essential to accommodate recovery, while practical modifications included changing delivery routes: *“I was on a van route, but it was a long day, and I asked to be put on a shorter route”* [24].

Adaptations often involved modifying the type of work undertaken. Post-surgical fatigue or physical limitations led some men to pursue less technical tasks or shift into different areas of the business. This allowed them to remain productive while managing their capabilities: *“I didn’t think I could do the technical stuff as well as I used to, and I didn’t want to take it easy when everybody else was working hard. So, I pondered about doing something else instead”* [25]. Opportunities to take on new responsibilities also supported ongoing engagement: *“I couldn’t work in the (machining) shop. Luckily, the owner was looking to expand the business … So, I told the owner I could do some marketing and help out that way. That’s how I began to learn about promoting our company and incorporated marketing into my job description”* [25].

Supportive workplaces were valued for providing flexibility and understanding. Survivors emphasised the importance of employers adapting responsibilities to side effects and allowing *“breathing room”* or *“a space to recover”* while at work [11]. Few men expressed a desire to change jobs, reflecting *sustained interest in their role, a belief that they were too old to change or that it would be difficult to obtain a new job following a cancer diagnosis* [9].

### Work identity, values, and priorities

Work held multifaceted significance for men following prostate cancer treatment. For some, it was central to their identity: *“The only way I know how to live my life is if I work and my work is my life”* [9]. Work also represented a sense of normality and continuity: *“I think work has a highly influentially symbolic role for a lot of people and it’s very closely associated with people’s sense of identity and worth … Certainly in my own case, I knew from the outset that I wanted this to be the shortest hiccup in my working life”* [9]. In this context, work could be therapeutic, offering a distraction from the illness and a challenge that prevented rumination: *“I think work was very therapeutic. I mean if I’d sat at home contemplating how terrible life was. That would be awful. I was very lucky to have, you know, a challenging job that kept me going”* [9]. Even during treatment, work served as a coping mechanism: *“I was going to work [during treatment]. I finished my external beam radiotherapy nine to nine‐fifteen [in the morning] and at ten o’clock, I am [working]. That was the way I could cope”* [22].

For others, work was framed more pragmatically, as *“a necessary evil”* [21] or a role sustained primarily for financial reasons, prompting reassessment of priorities: *“We never listen to our bodies. I learned the hard way. Now, I take half a day off to see the doctor, even if it is for a minor thing”* [25]. Following treatment, many men also reflected on life beyond work, expressing a desire to enjoy retirement: *“I don’t want to work forever. I’m not one of those people. I want to enjoy life. There are things that I’d like to do while I’m still able. I’d have no problems in retiring soon”* [25], and focus on family: *“I thought about this for a long time and felt that I should retire if the conditions (at work) were right. Instead, I could spend more time with my family”* [25].

These experiences illustrate how prostate cancer prompted a recalibration of priorities, balancing work commitments with health, family, and personal goals. Men emphasised positive attitudes: *“Surgery makes you see things differently. You are the same person up here (points at his head), but the body has changed. Up here (points at head), you wanna do the things you did, but your body says: ‘Nope. You can’t’. So, you work around that. You prioritize doing the things you can do and look for a happy medium”* [25] and determination as central to navigating post-surgical challenges: *“I didn’t give up. I kept telling myself: ‘I can’t let it win’”* [25]. Work, whether central to identity or sustained for practical reasons, remained a critical domain through which men negotiated recovery, resilience, and life post-treatment.

### Patterns across treatment types, age groups, and employment types

Some tentative patterns were observable across the included studies. Narratives concerning incontinence and its impact on workplace stigma were most prominent among men who had undergone radical prostatectomy [9–11, 25]. Fatigue and cognitive difficulties were described by treatment, with hormone therapy specifically linked to cognitive impacts on work in some accounts [21]. Self-employed men consistently described greater financial pressure and earlier-than-desired return to work compared with salaried employees, who more often had access to paid leave and graduated return arrangements [9, 21, 25]. Older men and those nearing retirement more commonly framed prostate cancer as prompting a reprioritisation toward health and family [20, 21, 25], whereas younger men more frequently emphasised work as central to identity and breadwinner roles [9, 23]. These observations should be interpreted as exploratory, given the heterogeneity of the included studies and the limited stratification of findings in primary reports.

## Discussion

We developed six analytical themes from 11 qualitative studies exploring work-related experiences of 302 men following prostate cancer treatment. Men’s return to work was characterised by variable trajectories. Financial pressures frequently dictated the urgency of returning to work. Workplace and family support facilitated reintegration, while treatment side effects introduced challenges, including stigma. Men employed a range of adaptations to accommodate ongoing health needs, and although work remained central to identity and coping for many, priorities often shifted post-treatment toward balancing health, family, and personal goals.

Men’s return-to-work experiences illustrate the complex interplay between health, occupational demands, and social context. The variability in return-to-work trajectories highlights that recovery cannot be understood solely in terms of physical healing, underscoring the need for personalised planning and realistic expectations, particularly for those in self-employment or precarious work. These findings align with research in other cancers showing that self-employment and precarious roles similarly constrain recovery and necessitate individualised support [26, 27]. These pressures may be particularly salient for prostate cancer survivors, who are generally older than those with other cancers and may be established in mid- or late-career roles, often combined with family responsibilities [28]. The age profile also means that some prostate cancer survivors may hold senior or leadership positions, where reduced capacity can have wider organisational impact. A one-size-fits-all approach to return-to-work planning therefore risks ignoring the trade-offs between identity, income, business continuity, and health management.

Treatment-related side effects, most notably incontinence, had profound implications for work performance and psychosocial wellbeing. Incontinence and the fear of social judgment required survivors to negotiate workplace disclosure, adapt work tasks, and manage stigma in real time. These challenges are intertwined with broader societal expectations of masculinity and the breadwinner role, particularly in older men who may feel pressure to maintain work performance despite health limitations [29]. Comparable findings have been reported in studies of other cancers and chronic illnesses, where treatment-related bodily changes contribute to self-consciousness, concealment behaviours, and social anxiety in the workplace [30–32]. Fatigue and cognitive limitations also restricted decision-making, task performance, and self-efficacy, pointing to the importance of structured, phased return to work. Many men were reluctant to change roles or jobs, often reflecting older age and perceived limited opportunities to retrain; if current roles cannot be adapted, this can translate into premature exit from the workforce. Advances in prostate cancer management including less invasive surgical approaches, shorter radiation courses, and improved side-effect management [33] could potentially reduce some functional limitations, though whether this translates into improved work outcomes remains uncertain and warrants future investigation.

The central role of support systems in facilitating work participation reinforces the interdependence of survivors’ personal and professional networks. Supportive employers, colleagues, family, and community resources not only mitigated practical challenges but also reinforced self-confidence, identity, and a sense of fulfilling masculine roles. Several practical implications follow from these findings. Clinicians could initiate conversations about work and potential treatment-related adjustments at the point of diagnosis, ideally as part of a written survivorship plan that incorporates work goals alongside clinical follow-up. Early involvement of occupational health teams may help translate survivors’ needs into workable modifications, including tailored phased return arrangements responsive to fluctuating recovery. Self-employed men may benefit from targeted financial support programmes and signposting to disability and welfare services. Across these recommendations, integrated survivorship care should address psychosocial and identity-related needs alongside physical recovery. Evidence regarding the effectiveness of interventions to improve work reintegration for cancer survivors remains inconclusive [34–36], highlighting the need for prospective intervention trials evaluating tailored workplace support strategies for prostate cancer survivors.

Our findings also illuminate the ways in which prostate cancer prompts reflection on identity, priorities, and life goals. While work often remained central to identity and a sense of normality, survivors frequently recalibrated their priorities, weighing health, family, and personal fulfilment against occupational demands. Consistent with role and identity theories, this recalibration is shaped by gendered expectations that position employment as a key marker of status and meaning, which can heighten stress and influence the timing or extent of work resumption [37, 38]. Interventions that integrate career counselling, psychosocial support, and survivorship planning could help clinicians and employers support survivors in navigating these choices and achieving meaningful reintegration. Evidence from breast and colorectal cancer survivors similarly indicates that attention to role identity and life priorities shapes long-term work satisfaction and quality of life [39, 40], suggesting that these insights have broad applicability.

This is the first systematic synthesis dedicated to qualitative research on working after prostate cancer treatment. The included studies provided rich insights into the lived experiences of survivors; however, several limitations should be acknowledged. Many studies included only small or heterogeneous samples, and while work emerged as a salient theme, few were designed with work as a primary focus. Although most included studies were rated at moderate risk of bias, these ratings primarily reflected under-reporting of researcher reflexivity and philosophical positioning rather than concerns about the validity of participants’ accounts. Confidence in the analytical themes was therefore considered broadly high, with all themes supported across multiple studies of varying quality, including those rated at low risk of bias. Reporting of occupation, industry, and employment characteristics was inconsistent across studies, limiting the depth of analysis possible by these factors. Newer or less commonly used treatments, such as focal therapy or stereotactic radiotherapy, were not represented in the included studies, with most samples reflecting men treated with established modalities such as radical prostatectomy, external beam radiotherapy, hormone therapy, or chemotherapy. Limited attention was also given to the influence of cultural context on work experiences. Most studies examined return to work retrospectively, with little exploration of proactive planning with clinicians or employers prior to treatment, and few studies considered the work experiences of long-term survivors living more than a decade beyond diagnosis. Additionally, the review was restricted to English-language studies, and all were conducted in high-income countries, limiting generalisability to low- and middle-income contexts. Future research should address these gaps by examining work experiences prospectively, across diverse occupational settings, treatment modalities, and survivorship timeframes, and in more varied cultural and economic contexts.

In summary, prostate cancer survivors’ work experiences after treatment are shaped by an interplay of treatment effects, financial pressures, employment type, and workplace and family support. Where work adaptations and integrated survivorship support are lacking, survivors risk prolonged absence or early exit from the workforce, underscoring the need for prospective and intervention-based research to identify strategies that enable sustainable and meaningful work participation.

## Supplementary Information

Below is the link to the electronic supplementary material.

Supplementary file1 (PDF 185 kb)

## Data Availability

The datasets generated during and/or analysed during the current study are available from the corresponding author on reasonable request.
