# Supplementary material for: Navigating work after prostate cancer treatment: a systematic review of qualitative research
Source: J Cancer Surviv. 2026 Jun 16;20(Suppl 2):2534–47. doi: 10.1007/s11764-026-02062-w (PMC13744520; doi:10.1007/s11764-026-02062-w)
Supplement: Supplementary file 1 [file 11764_2026_2062_MOESM1_ESM.pdf]

**Navigating work after prostate cancer treatment: a systematic review of qualitative research – Supplementary information.**

Journal: Journal of Cancer Survivorship

Authors: Dunya Tomic, Tessa Keegel, Sophie K Schellack, Lisa Abdel-Malek, Caron Jander, Supun T Hettige, Ewan MacFarlane, Jeremy Millar, Karen Walker-Bone

Correspondence: Dr Dunya Tomic, Monash Centre for Occupational and Environmental Health, 553 St Kilda Road, Melbourne VIC 3004, Australia; Dee.Tomic@monash.edu

**Supplementary Table 1: Search strategies for identifying qualitative research on men's experiences of work after prostate cancer treatment.**

| Database | Search date (coverage) | Search terms |
|---|---|---|
| CINAHL | 2025 May 27 | S1 MM "Prostatic Neoplasms"<br>S2 TX ((prostat* N3 (cancer* or neoplasm* or tumor* or tumour*)))<br>S3 S1 or S2<br>S4 MM "Job re-entry"<br>S5 MM employment<br>S6 ((employ* or unemploy* or work*) N5 (return* or suviv* or rehabilitat* or continu*))<br>S7 S4 or S5 or S6<br>S8 S3 and S7 |
| Embase | 2025 May 27 (1947–) | 1. exp prostate surgery/<br>2. exp prostate tumor/<br>3. (prostat* adj3 (cancer* or neoplasm* or tumor* or tumour*)).ti,ab, kw.<br>4. or/1-3<br>5. return to work/<br>6. exp employment/<br>7. ((employ* or unemploy* or work*) adj5 (return* or surviv* or rehabilitat* or continu*)).ti,ab,kw.<br>8. or/5-7<br>9. and/4,8<br>10. or/2-3<br>11. 8 and 10 |
| Ovid Medline | 2025 May 27 (1946–) | 1. Prostatectomy<br>2. Prostatic Neoplasms<br>3. (prostat* adj3 (cancer* or neoplasm*)).ti,ab,kw.<br>4. or/1-3<br>5. exp work/<br>6. exp employment/<br>7. ((employ* or work*) adj3 (return* or surviv* or rehabilitat* or contu*)).ti,ab,kw<br>8. or/5-7 |

| | | |
|---|---|---|
| | | 9. and/4,8<br>10. (Prostate cancer treatment and work: a scoping review).m_titl.<br>11. Work after prostate cancer: a systematic review.m_titl<br>12. "Work ability in prostate cancer survivors after radical prostatectomy.".m_titl<br>13. The only way I know how to live is to work.m_title<br>14. 10 or 11 or 12 or 13<br>15. 9 or 14 |
| PsycInfo | 2025 May 27 (1806–) | 1. return to work/<br>2. exp employment/<br>3. (employ* or unemploy* or work*) adj5 (return* or surviv* or rehabilitati* or continu*)).ti,ab,id.<br>4. or/1-3<br>5. prostate/<br>6. (prostat* adj3 (cancer* or neoplasm* or tumor* or tumour*)).ab,ti,id.<br>7. or/5-6<br>8. and/5,7<br>9. 1 or 3<br>10. 7 and 9 |
| Google Scholar | 2025 May 27 (First 50 references) | Return to work after prostate cancer treatment |

**Supplementary Table 2: PRISMA checklist.**

| Topic | Item # | Checklist item | Location where item is reported |
|---|---|---|---|
| Title | 1 | Identify the report as a systematic review. | p.1 |
| Abstract | 2 | See the PRISMA 2020 for Abstracts checklist. | p.1 |
| Rationale | 3 | Describe the rationale for the review in the context of existing knowledge. | p.2 |
| Objectives | 4 | Provide an explicit statement of the objective(s) or question(s) the review addresses. | p.2 |
| Eligibility criteria | 5 | Specify the inclusion and exclusion criteria for the review and how studies were grouped for the syntheses. | p.3 |
| Information sources | 6 | Specify all databases, registers, websites, organisations, reference lists and other sources searched or consulted to identify studies. Specify the date when each source was last searched or consulted. | p.3 |
| Search strategy | 7 | Present the full search strategies for all databases, registers and websites, including any filters and limits used. | Supp. Table 1 |
| Selection process | 8 | Specify the methods used to decide whether a study met the inclusion criteria of the review, including how many reviewers screened each record and each report retrieved, whether they worked independently, and if applicable, details of automation tools used in the process. | p.3 |

| | | | |
|---|---|---|---|
| Data collection process | 9 | Specify the methods used to collect data from reports, including how many reviewers collected data from each report, whether they worked independently, any processes for obtaining or confirming data from study investigators, and if applicable, details of automation tools used in the process. | p.3 |
| Data items | 10 | List and define all outcomes for which data were sought. Specify whether all results that were compatible with each outcome domain in each study were sought (e.g. for all measures, time points, analyses), and if not, the methods used to decide which results to collect. | p.3 |
| Study risk of bias assessment | 11 | Specify the methods used to assess risk of bias in the included studies, including details of the tool(s) used, how many reviewers assessed each study and whether they worked independently, and if applicable, details of automation tools used in the process. | p.4 |
| Effect measures | 12 | Specify for each outcome the effect measure(s) (e.g. risk ratio, mean difference) used in the synthesis or presentation of results. | p.3 |
| Synthesis methods | 13 | Describe the processes used to decide which studies were eligible for each synthesis (e.g. tabulating the study intervention characteristics and comparing against the planned groups for each synthesis (item #5)). | p.3 |
| Reporting bias assessment | 14 | Describe any methods used to assess risk of bias due to missing results in a synthesis (arising from reporting biases). | N/A |
| Certainty assessment | 15 | Describe any methods used to assess certainty (or confidence) in the body of evidence for an outcome. | N/A |
| Study selection | 16 | Describe the results of the search and selection process, from the number of records identified in the search to the number of studies included in the review, ideally using a flow diagram. | Figure 1 |
| Study characteristics | 17 | Cite each included study and present its characteristics. | Table 1 |
| Risk of bias in studies | 18 | Present assessments of risk of bias for each included study. | Supp. Table 6; Supp. Figure 1 |
| Results of individual studies | 19 | For all outcomes, present, for each study: (a) summary statistics for each group (where appropriate) and (b) an effect estimate and its precision (e.g. confidence/credible interval), ideally using structured tables or plots. | N/A |
| Results of syntheses | 20 | For each synthesis, briefly summarise the characteristics and risk of bias among contributing studies. | p.5 |
| Reporting biases | 21 | Present assessments of risk of bias due to missing results (arising from reporting biases) for each synthesis assessed. | N/A |
| Certainty of evidence | 22 | Present assessments of certainty (or confidence) in the body of evidence for each outcome assessed. | N/A |
| Discussion | 23a | Provide a general interpretation of the results in the context of other evidence. | p.11 |
| | 23b | Discuss any limitations of the evidence included in the review. | p.13 |
| | 23c | Discuss any limitations of the review processes used. | p.13 |
| | 23d | Discuss implications of the results for practice, policy, and future research. | p.13 |

| | | | |
|---|---|---|---|
| Registration and protocol | 24 | Provide registration information for the review, including register name and registration number, or state that the review was not registered. | p.3 |
| Support | 25 | Describe sources of financial or non-financial support for the review, and the role of the funders or sponsors in the review. | p.15 |
| Competing interests | 26 | Declare any competing interests of review authors. | p.15 |
| Availability of data, code and other materials | 27 | Report which of the following are publicly available and where they can be found: template data collection forms; data extracted from included studies; data used for all analyses; analytic code; any other materials used in the review. | p.15 |

**Supplementary Table 3: ENTREQ checklist.**

| **Item** | **Item #** | **Checklist item** | **Location where the item is reported** |
|---|---|---|---|
| Aim | 1 | State the research question the synthesis addresses | p.2 |
| Synthesis methodology | 2 | Identify the synthesis methodology or theoretical framework which underpins the synthesis, and describe the rationale for choice of methodology | p.4 |
| Approach to searching | 3 | Indicate whether the search was pre-planned (comprehensive search strategies to seek all available studies) or iterative (to seek all available concepts until they theoretical saturation is achieved). | p.4 |
| Inclusion criteria | 4 | Specify the inclusion/exclusion criteria (e.g. in terms of population, language, year limits, type of publication, study type). | p.3 |
| Data sources | 5 | Describe the information sources used and when the searches conducted; provide the rationale for using the data sources. | p.3 |
| Electronic search strategy | 6 | Describe the literature search (e.g. provide electronic search strategies with population terms, clinical or health topic terms, experiential or social phenomena related terms, filters for qualitative research, and search limits). | Supp. Table 1 |
| Study screening methods | 7 | Describe the process of study screening and sifting (e.g. title, abstract and full text review, number of independent reviewers who screened studies). | p.3 |
| Study characteristics | 8 | Present the characteristics of the included studies (e.g. year of publication, country, population, number of participants, data collection, methodology, analysis, research questions). | Table 1 |
| Study selection | 9 | Identify the number of studies screened and provide reasons for study exclusion (e.g. for comprehensive searching, provide numbers of studies screened and reasons for exclusion indicated in a figure/flowchart; for iterative searching describe reasons for study exclusion and inclusion based on modifications to the research question and/or contribution to theory development). | Figure 1 |

| | | | |
|---|---|---|---|
| Rationale appraisal | 10 | Describe the rationale and approach used to appraise the included studies or selected findings (e.g. assessment of conduct (validity and robustness), assessment of reporting (transparency), assessment of content and utility of the findings). | p.4 |
| Appraisal items | 11 | State the tools, frameworks and criteria used to appraise the studies or selected findings (e.g. Existing tools: CASP, QARI, COREQ, Mays and Pope [25]; reviewer developed tools; describe the domains assessed: research team, study design, data analysis and interpretations, reporting). | p.4 |
| Appraisal process | 12 | Indicate whether the appraisal was conducted independently by more than one reviewer and if consensus was required. | p.4 |
| Appraisal results | 13 | Present results of the quality assessment and indicate which articles, if any, were weighted/excluded based on the assessment and give the rationale. | Supp. Table 6; Supp. Figure 1 |
| Data extraction | 14 | Indicate which sections of the primary studies were analysed and how were the data extracted from the primary studies? | p.4 |
| Software | 15 | State the computer software used, if any. | p.4 |
| Number of reviewers | 16 | Identify who was involved in coding and analysis. | p.4 |
| Coding | 17 | Describe the process for coding of data (e.g. line by line coding to search for concepts). | p.4 |
| Study comparison | 18 | Describe how were comparisons made within and across studies (e.g. subsequent studies were coded into pre-existing concepts, and new concepts were created when deemed necessary). | p.4 |
| Derivation of themes | 19 | Explain whether the process of deriving the themes or constructs was inductive or deductive. | p.4 |
| Quotations | 20 | Provide quotations from the primary studies to illustrate themes/constructs, and identify whether the quotations were participant quotations or the author's interpretation. | p.5 |
| Synthesis output | 21 | Present rich, compelling and useful results that go beyond a summary of the primary studies (e.g. new interpretation, models of evidence, conceptual models, analytical framework, development of a new theory or construct). | p.5 |

**Supplementary Table 4: Data extraction template.**

| **Field** | **Description** |
|---|---|
| Author, year | First author surname and publication year |
| Source | How the study was identified (database search / reference checking) |
| Country | Country in which the study was conducted |
| Study aim/focus | Stated aim or primary focus of the study |
| Sample size | Number of participants with prostate cancer |
| Participant characteristics | Age (range, mean/median), ethnicity, employment status / type, occupation |
| Treatment details | Type(s) of prostate cancer treatment received (e.g., radical prostatectomy, radiotherapy, hormone therapy, chemotherapy, active surveillance, watchful waiting) |

| | |
|---|---|
| Data collection methods | Method (e.g., semi-structured interviews, focus groups), format (face-to-face, telephone), duration |
| Timing of data collection | Time of data collection relative to prostate cancer diagnosis or treatment |
| Qualitative analytic approach | Approach used to analyse qualitative data (e.g., framework analysis, inductive thematic analysis) |
| Work-related findings | All qualitative data relating to participants' work experiences during/after treatment, including verbatim quotes and author-reported themes |

**Supplementary Table 5: Worked example of the coding process for the analytical theme "Treatment side effects and stigma".**

| Original quote / extract | Descriptive code | Descriptive theme | Analytical theme |
|---|---|---|---|
| *"Mainly because I feel weak and tired and I don't feel like I can remember things as well as I used to, and I have the incontinence which is a pain to do while you're doing things. So yes, I'd say it would impact my work and things."* (Al Hussein Al Awamlh, 2024 [10]) | Fatigue and incontinence impaired work | Treatment side effects and their impact on work | Treatment side effects and stigma |
| *"The fatigue [is] like that, it's not just physical, because it affects your mental processes as well. After a while you find you can't think clearly, so you're not in the right space for making proper management decisions and that sort of thing. So that has a direct impact on your ability to work."* (Liu, 2023 [21]) | Fatigue impaired cognitive work performance | Treatment side effects and their impact on work | Treatment side effects and stigma |
| *"I don't feel like I was adequately informed around the impacts of treatment, particularly the hormone therapy … I didn't feel adequately prepared for the way the hormone therapy affected me … It affects your cognitive function so I felt like I couldn't [work]."* (Liu, 2023 [21]) | Hormone therapy impaired cognition, work ability | Treatment side effects and their impact on work | Treatment side effects and stigma |
| *"If you can imagine two or three blokes in a meeting, you take your comfort break, everyone trots off for a pee. They're all going to these appalling looking urinals that blokes use. I've got to trot into a cubicle and change a pad. It's kind of socially abnormal …"* (Grunfeld, 2013 [9]) | Incontinence stigma at work | Communication, disclosure, and stigma | Treatment side effects and stigma |
| *"You are thrown back into it and you wonder how you will be received, whether people will say 'Here he comes, he's been loafing off for three months.' So, they don't look at you, at what you've done and could do."* (Grunfeld, 2013 [9]) | Fear of stigma after absence | Communication, disclosure, and stigma | Treatment side effects and stigma |
| *"When you get around the individuals, their mouth drops … their eyes get big, and they look at you. I'll say, 'Yeah, okay, I've got cancer. And I've dealt with that issue.' Let's get over the… shock, [I'm] not damaged goods. I often felt like that when individuals would talk to me and look at me sometimes."* (Owens, 2024 [11]) | Radical prostatectomy survivor felt stigmatised at work | Communication, disclosure, and stigma | Treatment side effects and stigma |

**Supplementary Table 6: Risk of bias assessment of included studies using the Joanna Briggs Institute critical appraisal checklist for qualitative research.**

| Author, year | Q1 | Q2 | Q3 | Q4 | Q5 | Q6 | Q7 | Q8 | Q9 | Q10 | Overall risk of bias |
|---|---|---|---|---|---|---|---|---|---|---|---|
| Al Hussein Al | Unclear | Yes | Yes | Yes | Yes | No | No | Yes | Yes | Yes | Moderate |

| | | | | | | | | | | | |
|---|---|---|---|---|---|---|---|---|---|---|---|
| Awamlh, 2024 [10] | | | | | | | | | | | |
| Appleton, 2015 [18] | Yes | Yes | Yes | Yes | Yes | No | No | Yes | Yes | Yes | Low |
| Gray, 2005 [19] | Unclear | Yes | Yes | Yes | Yes | Yes | Unclear | Yes | No | Yes | Moderate |
| Grunfeld, 2013 [9] | Unclear | Yes | Yes | Yes | Yes | No | No | Yes | Unclear | Yes | Moderate |
| Joyce, 2024 [20] | Yes | Yes | Yes | Yes | Yes | No | No | Yes | Yes | Yes | Low |
| Liu, 2023 [21] | Unclear | Yes | Yes | Yes | Yes | No | No | Yes | Yes | Yes | Moderate |
| Owens, 2024 [11] | Unclear | Yes | Yes | Yes | Yes | No | No | Yes | No | Yes | Moderate |
| Wagland, 2020 [22] | No | Yes | Yes | Yes | Yes | No | No | Yes | Yes | Yes | Moderate |
| Wennick, 2017 [23] | Unclear | Yes | Yes | Yes | Yes | No | No | Yes | Yes | Yes | Moderate |
| Yarker, 2010 [24] | Unclear | Yes | Yes | Yes | Yes | No | No | Yes | Unclear | Yes | Moderate |
| Yu Ko, 2020 [25] | Unclear | Yes | Yes | Yes | Yes | No | No | Yes | Yes | Yes | Moderate |

*Q1: Is there congruity between the stated philosophical perspective and the research methodology? Q2: Is there congruity between the research methodology and the research question or objectives? Q3: Is there congruity between the research methodology and the methods used to collect data? Q4: Is there congruity between the research methodology and the representation and analysis of data? Q5: Is there congruity between the research methodology and the interpretation of results? Q6: Is there a statement locating the researcher culturally or theoretically? Q7: Is the influence of the researcher on the research, and vice-versa, addressed? Q8: Are participants, and their voices, adequately represented? Q9: Is the research ethical according to current criteria or, for recent studies, and is there evidence of ethical approval by an appropriate body? Q10: Do the conclusions drawn in the research report flow from the analysis, or interpretation, of the data?*

**Supplementary Figure 1: Detailed risk of bias results.**

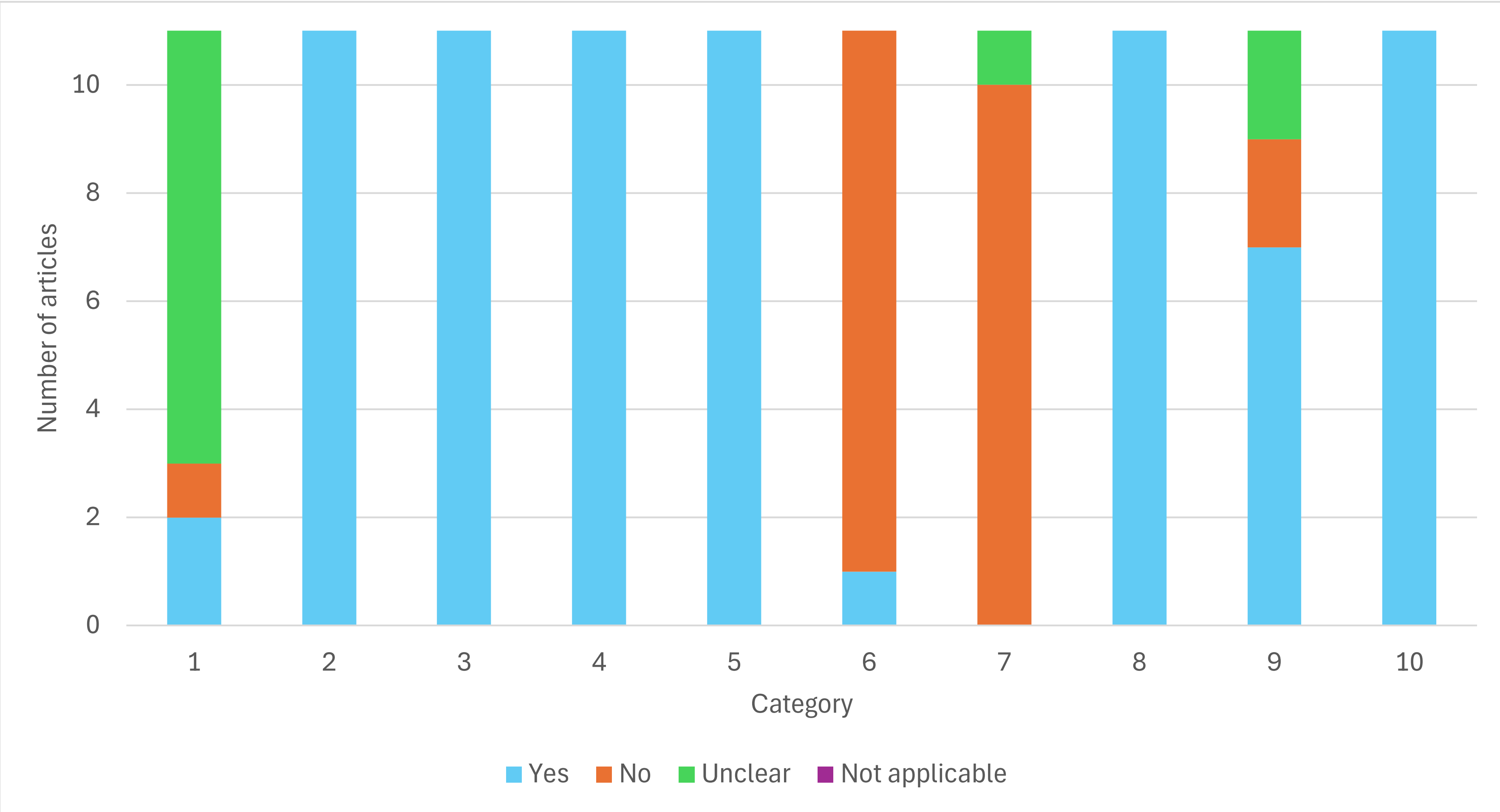

*See Supplementary Table 6 for full domain descriptions.*
